# Height and Risk of Gestational Diabetes Mellitus: Results from the Healthy Baby Cohort Study

**DOI:** 10.1155/2018/4679245

**Published:** 2018-08-07

**Authors:** Hui Li, Lulu Song, Lijun Shen, Bingqing Liu, Xiaoxuan Zheng, Lina Zhang, Youjie Wang, Zhongqiang Cao, Shunqing Xu

**Affiliations:** ^1^Department of Maternal and Child Health, School of Public Health, Tongji Medical College, Huazhong University of Science and Technology, Wuhan, Hubei 430030, China; ^2^Wuhan Children's Hospital (Wuhan Maternal and Child Healthcare Hospital), Tongji Medical College, Huazhong University of Science and Technology, Wuhan, Hubei 430030, China; ^3^Key Laboratory of Environment and Health, Ministry of Education & Ministry of Environmental Protection, and State Key Laboratory of Environmental Health, School of Public Health, Tongji Medical College, Huazhong University of Science and Technology, Wuhan, Hubei 430030, China

## Abstract

**Background:**

The aim of this study was to examine the association between height and plasma glucose level, as well as risk of GDM among Chinese women.

**Methods:**

A total of 6941 pregnant Chinese women were recruited from the Healthy Baby Cohort study in Hubei Province, China, in 2012–2014. Measured height was categorized into four groups according to the quartile distribution (≤158.0 cm, 158.1–161.0 cm, 161.1–164.0 cm, and >164.0 cm). GDM was defined based on the International Association of the Diabetes in Pregnancy Study Group criteria. Linear regression was used to estimate the association between height and plasma glucose levels. Logistic regression was used to calculate odds ratios (ORs) and 95% confidence intervals (CIs) for the association between height and GDM.

**Results:**

The prevalence of GDM was 14.7% in our study. Height was inversely associated with the 1 h and 2h plasma glucose levels (all *P* value for trend < 0.05), but not with fasting plasma glucose levels. A significant negative trend was found between height and risk of GDM (*P* value for trend < 0.05), and each centimeter increase in height was associated with 2% (OR: 0.98; 95% CI: 0.96, 0.99) lower risk of GDM. Women in the highest quartile of height (>164.0 cm) had 23% (OR: 0.77; 95% CI: 0.64, 0.94) lower risk of developing GDM than those in the shortest quartile of height (≤158.0 cm), after adjusting for potential confounders.

**Conclusions:**

Our findings suggested that height was negatively associated with risk of GDM among Chinese women. The difference in plasma glucose levels is present in the 1 h and 2 h plasma glucose, but not with fasting plasma glucose.

## 1. Introduction

Gestational diabetes mellitus (GDM) is characterized by glucose intolerance with onset or first recognition during pregnancy [1]. The prevalence of GDM ranged from 9.3% to 25.5% among 15 collaborating centers using the International Association of the Diabetes and Pregnancy Study Group (IADPSG) criteria [2]. GDM is not only related to adverse pregnancy outcomes [3] but it is also associated with adverse long-term health effects on both mothers and their offspring [4, 5]. Identification of women at risk of GDM may allow early health monitoring and intervention.

Adult height is determined by the combination of genetic and environmental factors [6]. Studies have suggested that height was negatively associated with glucose tolerance in adults [7, 8]. Furthermore, a meta-analysis has demonstrated that shorter height was associated with the development of type 2 diabetes [9]. Given that GDM and type 2 diabetes share several similar pathogenic processes [10, 11], it is plausible that shorter height may also increase the risk of GDM.

Previous studies have shown an inverse relationship between height and risk of GDM in several countries [7, 12–15], but not all [16, 17]. However, due to the variations in height among different ethnic and socioeconomic groups [14], their results might not generalize to Chinese women. In addition, the different diagnostic criteria for GDM may affect the results. To our knowledge, the association between height and risk of GDM using the International Association of the Diabetes in Pregnancy Study Group (IADPSG) criteria has not been previously investigated in the Chinese population.

Therefore, we examined the association between height and plasma glucose level, as well as risk of GDM among pregnant Chinese women based on data from the Healthy Baby Cohort (HBC) study.

## 2. Methods

### 2.1. Study Participants

The HBC study was conducted at the Women and Children Medical and Healthcare Center of Wuhan City in the Hubei Province, China. This ongoing prospective cohort study is designed to assess the environmental and genetic factors that affect child health and development. Briefly, a total of 11,311 pregnant women who gave birth at this hospital were recruited between September 2012 and October 2014. Each participant was required to provide blood and urine samples and complete a standard questionnaire by a face-to-face interview at the time of institutional delivery. In the present study, we excluded participants with a history of diabetes (*n* = 6), those with missing information on height or other important covariates (*n* = 70), or those with missing values for fasting, 1 h, and 2 h plasma glucose (*n* = 4294). The final analytic sample included 6941 women. Except for alcohol consumption before pregnancy, the main characteristics of the women with OGTT results (6941) and the women without OGTT results (4294) on height (161.26 ± 4.52 cm versus 161.06 ± 4.54 cm), age at delivery (28.48 ± 3.46 years versus 27.77 ± 4.01 years), prepregnancy BMI (20.69 ± 2.75 kg/m^2^ versus 20.27 ± 2.59 kg/m^2^), education level (college or above rate: 73.6% versus 76.5%), employment status (employed rate: 84.4% versus 74.7%), exposure to passive smoking during pregnancy (88.3% versus 86.2%), smoking before pregnancy (0.5% versus 1.1%), and taking physical activity (yes: 87.7% versus 89.4%) were statistically different.

The study was approved by the Medical Ethics Committee of the School of Public Health, Tongji Medical College, Huazhong University of Science and Technology. All participants provided written informed consent at recruitment.

### 2.2. Assessment of Height

The height was measured without shoes, using a stadiometer, and recorded to the nearest 0.1 centimeter (cm) during the first antenatal care visit in the hospital. We categorized height into four groups according to the quartile distribution (≤158.0 cm, 158.1–161.0 cm, 161.1–164.0 cm, and >164.0 cm).

### 2.3. Diagnosis of GDM

In this study, all pregnant women underwent a 75 g 2 h oral glucose tolerance test (OGTT) in the morning after overnight fasting of at least 8 hours at 24–28 gestational weeks. Fasting, 1 h, and 2 h plasma glucose levels were measured at the Women and Children Medical and Healthcare Center of Wuhan City using the Roche Modular P800 Automatic Biochemistry Analyzer. According o the IADPSG criteria [18], women were diagnosed with GDM if they met any of the following cutoff points: fasting plasma glucose ≥ 5.1 mmol/l, 1 h plasma glucose ≥ 10.0 mmol/l, or 2 h plasma glucose ≥ 8.5 mmol/l.

### 2.4. Assessment of Covariates

Data on demographic characteristics (maternal age, educational level, and employment status) and lifestyle factors (alcohol consumption before pregnancy, smoking before pregnancy, passive smoking during pregnancy, and physical activity during pregnancy) were collected by questionnaires. Passive smoking was defined as exposure second-hand smoking more than once per week and for >15 min per time. Information on parity was obtained from medical records. Prepregnancy weight was self-reported at the first antenatal care visit (usually at the first trimester). Prepregnancy body mass index (BMI) was calculated as self-reported prepregnancy weight in kilograms divided by the measured height in square meters.

### 2.5. Statistical Analysis

Continuous variables were described as mean ± standard deviation (SD), and categorical variables were presented as a number and percentage. To compare the differences between the GDM group and non-GDM group, Student's *t*-test was used for continuous variables, and chi-square test was used for categorical variables.

Differences in mean fasting, 1 h, and 2 h plasma glucose levels among height quartiles were compared by analysis of covariance. Dunnett's test was used for post hoc analysis. Linear regression models were used to estimate the association of fasting, 1 h, and 2 h plasma glucose levels with height. Logistic regression models were used to estimate odds ratios (ORs) and 95% confidence intervals (CIs) for the association between height and risk of GDM. Models were fit using height as a categorical variable, based on the quartile distribution of height, and the lowest quartile was used as the reference group. Covariates was selected based on established or potential associations with GDM [4, 5], including maternal age (continuous), prepregnancy BMI (continuous), educational level (high school or below, college or above), employment status (employed or unemployed), parity (primiparous or multiparous), alcohol consumption before pregnancy (yes or no), smoking before pregnancy (yes or no), passive smoking during pregnancy (yes or no), and physical activity frequency during pregnancy (never/rarely, 1-2 days/week, 3-4 days/week, and 5-6 days/week or daily). In order to test for linear trend between height and plasma glucose levels or risk of GDM, we estimated the statistical significance by assigning the median values of each quartile of maternal height and fitting this as a continuous variable in a separate regression model.

To assess a potential effect modification, we performed subgroup analyses to examine the association between height and risk of GDM stratified by maternal age (<28 or ≥28 years, the median value of age at delivery), prepregnancy BMI (<24 or ≥ 24 kg/m^2^, the cutoff value of overweight for Chinese adults [19]), educational level (high school or below, college or above), and parity (primiparous or multiparous). Tests for interaction across subgroup were conducted using the Wald test.

All analyses were performed using SAS 9.4 software (SAS Institute Inc., Cary, NC, USA). A two-tailed *P* value < 0.05 was considered statistically significant.

## 3. Results

Among the 6941 women, the mean age at delivery was 28.5 (SD: 3.5) years, the mean height was 161.3 (SD: 4.5) cm, and 89.6% were primiparous. In total, 1017 (14.7%) women were diagnosed with GDM based on the IADPSG criteria used in the present study. Differences in distribution of the characteristics between GDM group and non-GDM group are presented in Table 1. Women with GDM were older and shorter, had lower educational level and higher prepregnancy BMI, and were more likely multiparous (all *P* values < 0.05).

Table 2 shows the mean fasting, 1 h, and 2 h plasma glucose levels according to height quartiles. Mean 1 h and 2 h plasma glucose levels decreased linearly with increasing height (all *P* values for trend < 0.001) without adjustment for confounders. After adjustment for potential confounders, the height was still inversely associated with the 1 h and 2 h plasma glucose levels (all *P* values for trend < 0.001). Post hoc analyses suggested that women with taller height had lower 1 h and 2 h plasma glucose levels than those with shorter height (158.1–161.0 cm versus ≤158.0 cm, *P* < 0.05; 161.1–164.0 cm versus ≤158.0 cm, *P* < 0.05; >164.0 cm versus ≤158.0 cm, *P* < 0.05).

Table 3 presents the relationship between height and fasting, 1 h, and 2 h plasma glucose levels. Linear associations of height with 1 h and 2 h plasma glucose levels were identified (all *P* values < 0.001). After controlling for potential confounders, every centimeter increase in height was associated with a linear change in 1 h plasma glucose levels with −0.0157 mmol/l (95% CI: −0.0240, −0.0075) and in 2 h plasma glucose levels with −0.0231 mmol/l (95% CI: −0.0297, −0.0165). No significant association was observed between height and fasting plasma glucose levels before and after adjusting for potential confounding variables.

**Figure 1:**
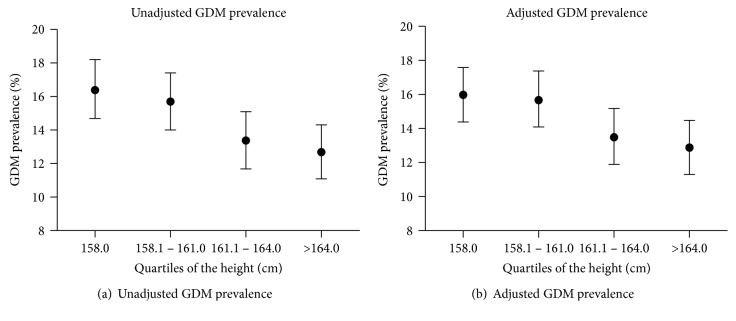
Unadjusted and adjusted gestational diabetes mellitus (GDM) prevalence according to height quartiles. (a) Association between height and unadjusted prevalence of GDM; (b) association between height and prevalence of GDM after adjusting for maternal age, prepregnancy BMI, educational level, employment status, parity, alcohol consumption before pregnancy, smoking before pregnancy, passive smoking during pregnancy, and physical activity during pregnancy. The vertical bars are 95% confidence intervals.

The unadjusted and adjusted prevalence of GDM according to height quartiles are shown in Figure 1. There was a descending linear relationship between height and prevalence of GDM before and after adjustment for potential confounders (*P* value for trend < 0.05).

Table 4 shows the unadjusted and adjusted ORs and 95% CIs for GDM according to height quartiles. A significant negative trend was found between height and risk of GDM (*P* value for trend < 0.05), and each centimeter increase in height was associated with 2% (OR: 0.98; 95% CI: 0.96, 0.99) lower risk of GDM. After adjustment for potential confounders, women in the highest quartile of height (>164.0 cm) had lower risk of GDM with an adjusted OR of 0.77 (95% CI: 0.64, 0.94), compared with those in the shortest quartile height (≤158.0 cm).

### 3.1. Subgroup Analyses

**Figure 2:**
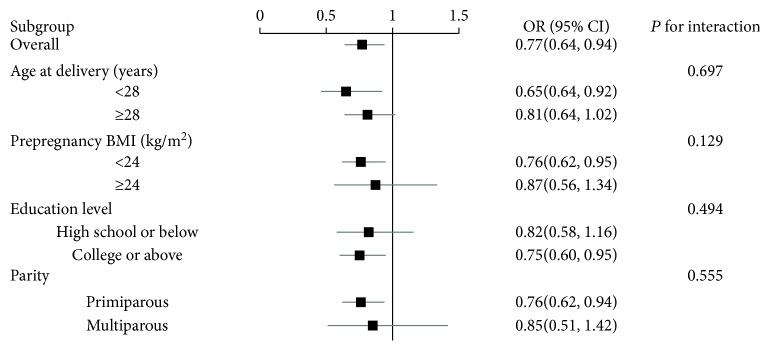
Subgroup analysis of associations between the highest quartile of height and gestational diabetes mellitus (GDM). Odds ratios for GDM are comparison of the highest quartile of height with the shortest quartile of height. Analyses were adjusted for maternal age, prepregnancy BMI, educational level, employment status, parity, alcohol consumption before pregnancy, smoking before pregnancy, passive smoking during pregnancy, and physical activity during pregnancy. Horizontal lines are 95% confidence intervals.

The inverse relationship between height and risk of GDM was generally similar across the subgroup stratified by maternal age, prepregnancy BMI, educational level, and parity (all *P* values for interaction > 0.05) (Figure 2).

## 4. Discussion

In this study, we investigated the association between height and risk of GDM among Chinese women. We observed height was negatively associated with risk of GDM after adjustment for potential confounders. In addition, we found that height was inversely associated with the 1 h and 2 h plasma glucose levels, but not with fasting plasma glucose levels.

Several studies have investigated the association between height and GDM, and the findings were inconsistent. The Omega Cohort Study including 1644 American women suggested an inverse relationship between self-reported height and risk of GDM based on the National Diabetes Data Group (NDDG) diagnostic criteria independent of age, race/ethnicity, education, and prepregnancy BMI [12]. Similarly, the relationship between height and risk of GDM using the NDDG criteria was observed in a study of 2772 Greek women and a study of 9005 Korean women [7, 13]. A study recruited 126,861 American women and shown that the risk of GDM decreased linearly with increasing height using the American Diabetes Association Criteria [14]. However, in their study, height data were measured or self-reported. Branchtein et al. investigated 4973 Brazilian women and found a negative association between height and GDM using the 1988 World Health Organization (WHO) criteria [15]. In contrast, a study of 1635 women from Hungary reported no significant association between height and risk of GDM according to the 1998 WHO criteria [16]. The DALI study including 971 women found that decreased height was associated with significantly decreased risk of GDM using the IADPSG criteria [17]. These discrepancies across these studies may be due to the differences in height among ethnic groups, diagnostic criteria for GDM, race/ethnicity, and sample size.

The mechanisms underlying the relationship between height and risk of GDM are not clear. GDM is characterized by pancreatic *β*-cell dysfunction and insulin resistance [10]. Shorter adult stature predicts impaired *β*-cell function and insulin resistance [7, 20], which was associated with an increased risk of GDM. In addition, intrauterine nutrition and childhood nutrition are important determinants of adult height [6, 21]. Inadequate intrauterine and childhood nutrition might increase risk of diabetes during adulthood [22–24]. Our study had no information on intrauterine and childhood nutrition, which prevented us from exploring whether height or nutrition has an effect on GDM. Further studies are needed to investigate the association between height and risk of GDM independent of intrauterine and childhood nutrition.

Low socioeconomic status during childhood was reported to be related with shorter adult height [25]. Studies have suggested that individuals who grew up in low socioeconomic status were more likely to develop diabetes in later life [26, 27]. Given that educational level is considered as a reliable marker of socioeconomic status during childhood [28], we adjusted educational level in our study and found that the significant association between height and risk of GDM still remained. In addition, we performed a subgroup analysis according to educational level and found that the inverse association between height and risk of GDM was consistent across the subgroup stratified by educational level. Therefore, our results suggested that the association between height and risk of GDM could not be fully explained by socioeconomic status during childhood.

Interestingly, we observed that height was inversely correlated with 1 h and 2 h plasma glucose levels, but not with fasting plasma glucose levels. Our findings were in agreement with a study from Brazil, reporting a negative association of height with 1 h and 2 h plasma glucose levels but not with fasting glucose levels without adjusting any [15]. Similarly, two studies conducted in Australia and Poland suggested that shorter women have higher 2 h plasma glucose levels than taller women, but not fasting plasma glucose levels [29, 30].

We found height was negatively associated with 1 h and 2 h plasma glucose, but not with fasting plasma glucose. A possible reason for this may be the different metabolisms of a standard dose of glucose during OGTT among taller and shorter women. Women with shorter stature have a lower mass of metabolically active tissues in response to a 75 g OGTT compared with women with taller stature [31].

Our study had several strengths, including a large sample size and a wide range of potential confounders. In addition, the diagnosis of GDM in our study was based on the measure of OGTT, performed with unified instruments at one hospital, which minimized the misclassification of GDM and non-GDM cases.

However, some limitations should be considered. First, some characteristics of the women with OGTT results and without OGTT results were statistically different, which might introduce selection bias, potentially reducing the generalization of this analysis. However, the differences were relatively small. Due to the large sample of the women with OGTT results (6941) and the women without OGTT results (4294), a slight difference would lead to a *P* value < 0.05. Moreover, the participants did not know the research purpose at the stage of data collecting; it was unlikely that women with shorter height tended to take or not take the OGTT test in this study. Second, although a variety of known risk factors of GDM were taken into account in the analyses of our study, it is not possible to rule out the residual confounding attributable to unmeasured factors, such as early-life nutrition factors, history of GDM, family history of diabetes, and maternal dietary intakes during pregnancy. Third, height and GDM might be jointly determined by genetic factors, but we are unable to examine their association.

## 5. Conclusion

Our result suggested that height was negatively associated with risk of GDM in pregnant Chinese women, independent of potential confounders. The difference in plasma glucose levels is present in the 1 h and 2 h plasma glucose, but not with fasting plasma glucose.

## Figures and Tables

**Table 1:** Characteristics of the study participants according to occurrence of gestational diabetes mellitus.

	GDM	Non-GDM	*P* value
Number of participants	1017	5924	—
Age at delivery (years)	29.56 ± 3.88	28.30 ± 3.35	<0.001^a^
Height (cm)	160.80 ± 4.63	161.34 ± 4.50	<0.001^a^
Prepregnancy BMI (kg/m^2^)	21.52 ± 3.08	20.55 ± 2.66	<0.001^a^
Educational level			<0.001^b^
High school or below	319 (31.4)	1513 (25.5)	
College or above	698 (68.6)	4411 (74.5)	
Employment status			0.006^b^
Employed	829 (81.5)	5030 (84.9)	
Unemployed	188 (18.5)	894 (15.1)	
Parity			<0.001^b^
Primiparous	854 (84.0)	5365 (90.6)	
Multiparous	163 (16.0)	559 (9.4)	
Alcohol consumption before pregnancy			0.872^b^
Yes	21 (2.1)	127 (2.1)	
No	996 (97.9)	5797 (97.9)	
Smoking before pregnancy			0.263^b^
Yes	8 (0.8)	30 (0.5)	
No	1009 (99.2)	5894 (99.5)	
Passive smoking during pregnancy			0.461^b^
Yes	112 (11.0)	700 (11.8)	
No	905 (89.0)	5224 (88.2)	
Physical activity during pregnancy			0.510^b^
Never or rarely	105 (10.3)	631 (10.7)	
1-2 days/week	82 (8.1)	556 (9.4)	
3-4 days/week	83 (8.2)	426 (7.2)	
5-6 days/week	13 (1.3)	90 (1.5)	
Daily	734 (72.2)	4221 (71.3)	

Data are mean ± SD or numbers (percentages). BMI: body mass index; GDM: gestational diabetes mellitus. ^a^Derived from Student's *t*-test. ^b^Derived from chi-square test.

**Table 2:** Mean plasma glucose levels according to height quartiles.

Plasma glucose (mmol/l)	Height quartiles (cm)				*P* for trend^b^
	≤158.0 *n* (1936)	158.1–161.0 *n* (1789)	161.1–164.0 *n* (1567)	>164.0 *n* (1649)	
Unadjusted					
Fasting	4.44 ± 0.50	4.45 ± 0.53	4.44 ± 0.50	4.47 ± 0.50	0.253
1 h	7.36 ± 1.63	7.29 ± 1.67	7.22 ± 1.63^a^	7.12 ± 1.59^a^	<0.001
2 h	6.63 ± 1.36	6.51 ± 1.30^a^	6.40 ± 1.28^a^	6.34 ± 1.21^a^	<0.001
Adjusted for age, prepregnancy BMI, educational level, occupational status, parity, alcohol consumption before pregnancy, smoking before pregnancy, passive smoking during pregnancy, and physical activity during pregnancy					
Fasting	4.44 ± 0.48	4.45 ± 0.51	4.44 ± 0.51	4.47 ± 0.49	0.131
1 h	7.34 ± 1.58	7.29 ± 1.57	7.23 ± 1.58^a^	7.14 ± 1.58^a^	<0.001
2 h	6.61 ± 1.28	6.51 ± 1.27^a^	6.41 ± 1.27^a^	6.35 ± 1.26^a^	<0.001

Data are mean ± SD. ^a^*P* < 0.05 for the comparison with the shortest quartile of height by using analysis of covariance and Dunnett's test for post hoc analysis. ^b^*P* values for trend were performed by assigning the median values of each quartile of height and fitting this as a continuous variable in a separate regression model.

**Table 3:** Regression coefficients (95% CIs) of plasma glucose levels for every centimeter increase in height.

Plasma glucose (mmol/l)	Unadjusted regression coefficient^a^ (95% CI)	*P* value	Adjusted regression coefficient^b^ (95% CI)	*P* value
Fasting	0.0018 (−0.0009, 0.0045)	0.184	0.0023 (−0.0003, 0.0049)	0.087
1 h	−0.0197 (−0.0282, −0.0112)	<0.001	−0.0157 (−0.0240, −0.0075)	<0.001
2 h	−0.0256 (−0.0324, −0.0189)	<0.001	−0.0231 (−0.0297, −0.0165)	<0.001

CI: confidence intervals. ^a^Not adjusted for any confounders. ^b^Adjusted for maternal age, prepregnancy BMI, educational level, employment status, parity, alcohol consumption before pregnancy, smoking before pregnancy, passive smoking during pregnancy, and physical activity during pregnancy.

**Table 4:** ORs (95% CIs) for the association between height and gestational diabetes mellitus.

Height quartiles (cm)	Number of participants	Number of GDM cases	Unadjusted OR^a^ (95% CI)	Adjusted OR^b^ (95% CI)
≤158.0	1936	317	1.00 (reference)	1.00 (reference)
158.1–161.0	1789	281	0.95 (0.80, 1.13)	0.98 (0.82, 1.17)
161.1–164.0	1567	210	0.79 (0.65, 0.96)	0.82 (0.67, 0.99)
>164.0	1649	209	0.74 (0.61, 0.90)	0.77 (0.64, 0.94)
Per 1 cm increase in height	—	—	0.97 (0.96, 0.99)	0.98 (0.96, 0.99)
*P* values for trend^c^	—	—	<0.001	0.002

OR: odds ratio; CI: confidence intervals; GDM: gestational diabetes mellitus. ^a^Not adjusted for any confounders. ^b^Adjusted for maternal age, prepregnancy BMI, educational level, occupational status, parity, alcohol consumption before pregnancy, smoking before pregnancy, passive smoking during pregnancy, and physical activity during pregnancy. ^c^*P* values for trend were performed by assigning the median values of each quartile of maternal height and fitted this as a continuous variable in a separate regression model.

## Data Availability

Data are unsuitable for public deposition due to ethical restriction and privacy of participant data. Data are available from the Healthy Baby Cohort study on reasonable request for any interested researcher who meets the criteria for access to confidential data. The corresponding author (Youjie Wang) may be contacted to request data.
